# Using an integrated social cognition model to identify the determinants of QR code check-in compliance behaviors in the COVID-19 pandemic

**DOI:** 10.1177/13591053231209880

**Published:** 2023-11-08

**Authors:** Thi Nhung Mac, Daniel J Phipps, Joy Parkinson, Mandy Cassimatis, Kyra Hamilton

**Affiliations:** 1 Griffith University, Australia; 2 University of Jyväskylä, Finland; 3 Australian Catholic University, Australia; 4 University of California, Merced, USA

**Keywords:** health behavior, QR code check-in, social cognition theory, theory of planned behavior

## Abstract

In Australia, checking in while entering venues was a legal requirement during the COVID-19 pandemic to track potential infection sites. This two-wave correlational study used an integrated theory of planned behavior model including moral norms, anticipated regret, and habit to predict check-in compliance in a sample of 181 Victorians (Mean Age = 41.88, 56.4% female) and 162 Queenslanders (Mean Age = 43.26, 47.5% female). Habit and intention predicted behavior, while perceived behavioral control did not. Intention was predicted by baseline habit, attitude, subjective norm, and moral norm in the Victorian sample, while only baseline habit and moral norm predicted intention in the Queensland sample. This study has potential implications for reviewing previous strategies and for future pandemic preparedness, both by identifying the drivers of infection control compliance, and through the discussion of how differences in effects between states may be linked to each state’s experience of the pandemic (e.g. infection rates, lockdown length).

## Introduction

The highly contagious nature of the Coronavirus disease (COVID-19), an infectious disease caused by the SARS-CoV-2 virus, presented an unprecedented challenge to the health systems and economy globally (United Nations, 2020). From February 2023, deaths from COVID-19 have surpassed 6.9 million (World Health Organization, 2023). In an effort to minimize the spread of the COVID-19 pandemic, governments and health authorities implemented a variety of strategies to “flatten the curve.” That is, to reduce both the total number of infections and peak infection rate. Early examples of these strategies included large scale restrictions on movement such as shelter-in-place orders, curfews, and lockdowns (Czeisler et al., 2021; Grout et al., 2021), often paired with other non-pharmaceutical strategies such as mandated social distancing, face mask requirements, and encouraged hand sanitizer use (MacIntyre et al., 2021; Payne et al., 2022). However, as the pandemic continued, many of these strategies, particularly those restricting movement or placing density limits on businesses and public spaces, were viewed as heavy handed and a barrier to a strong economic recovery (ABS, 2020). As the initial peak of the pandemic passed and experts learned more about the nature of the virus, governments around the world sought to employ methods of controlling the spread of infections while moderating the effects of public health measures on businesses, supply chains, and daily life.

Starting from the early pandemic stages until widespread immunity or resistance to severe COVID-19 infection was achieved (i.e. through a 95% nation-wide vaccination rate and/or previous infection), efforts in Australia were centered around tracking and isolating potential exposures to the virus to inhibit uncontrolled community outbreaks. Central to this was the use of mandated and compulsory venue check-ins to rapidly identify and inform those who were likely exposed to the COVID-19 virus (Davies et al., 2023). For the most part, these systems were based upon using smartphones to scan a “Quick Response Code” or QR code upon entering a venue or business (DensoWave, 2022). Data from these QR code check-ins was then made available to state and territory health officials to rapidly identify COVID-19 exposure sites and notify primary close contacts to monitor their symptoms, self-isolate, quarantine, or get tested (Australian Government. Department of Health and Aged Care, 2022). In Australia, the use of QR code check-ins as a method of tracing and, thus, potentially controlling COVID-19 outbreaks was adopted on a national scale, with check-ins required in all venues including workplaces, schools, and retail businesses across all states and territories in Australia (SafeWorkAustralia, 2020). Over the course of the pandemic, this process became increasingly streamlined, as state governments established dedicated check-in apps, QR code posters were displayed at the entry of every premise, and businesses endeavored to verify visitors’ check-in as a requirement for service (SafeWorkAustralia, 2020).

Despite legal mandates and the high value placed on QR code tracking for controlling the COVID-19 pandemic by the Australian Government (Abbas et al., 2021), engagement and enforcement of the behavior were often reported as inconsistent (ABC News, 2021b). Such a pattern of sub-optimal compliance has been reflected in other infection control behaviors including vaccination, mask wearing, and self-isolation (Farooq et al., 2020; Floyd et al., 2022; Thaker, 2021). In part, it is possible the unsatisfactory levels of compliance to COVID-19 preventive behaviors, like QR code check-in requirements, is due to the lack of understanding of the mechanisms that underpin the behavior, and the associated effects this lack of understanding may have on public health messaging. Thus, given the potential value of QR code check-ins and its effectiveness in curbing infection rates, it seems warranted to identify the determinants of QR code check-in compliance behavior to gain knowledge on the social psychological factors that are associated with this behavior, especially given new variants of COVID-19 that are being identified or even the potential of an entirely new pandemic to emerge.

A key model for understanding human behaviors, including COVID-19 related preventive behavior, is the theory of planned behavior (Ajzen, 1991). The elegance of the theory of planned behavior lies in its parsimony which posits intention as the most proximal determinant of behavior, and intention as being predicted by social cognition beliefs surmised as attitude, subjective norm, and perceived behavioral control (Ajzen, 1991). Attitude toward the behavior illustrates the degree to which an individual believes the behavior will result in positive or negative outcomes or affective states (Ajzen, 1991). Subjective norm describes whether an individual believes significant others would approve or disapprove of them undertaking the behavior (Ajzen, 1991). Perceived behavioral control reflects the perceived ability to perform or overcome barriers to performing a behavior, as is also proposed to directly predict behavior (Ajzen, 1991). Generally, the theory of planned behavior hypothesizes when someone views a behavior with a more favorable attitude, more likely to be approved of by important others, and under their control, they are more likely to form an intention to perform that behavior and, in turn, act upon their formed intention (Ajzen, 1991).

Consistently, the theory of planned behavior has been shown to predict a modest portion of variance in health-related intentions and behaviors (Hamilton et al., 2020; McEachan et al., 2011). In the context of the COVID-19 pandemic, researchers have applied the model to identify potentially modifiable determinants of government recommended preventive behaviors including social distancing (Hagger et al., 2020), facial mask wearing (Pan and Liu, 2022), hand hygiene practices (Smith et al., 2022), and vaccination uptake (Hagger and Hamilton, 2022), with effects shown to hold across time (Kwok et al., 2021; Lao et al., 2023) and meta-analytic evidence supporting model constructs in predicting intentions and behaviors for COVID-19 preventive measures (Fischer and Karl, 2022). However, despite evidence in favor of the model, some scholars have critiqued the theory of planned behavior, positing that given the modest effect sizes often found, it is unlikely the model presents a complete set of predictors for either intention or behavior (Sniehotta et al., 2014).

One example of a construct that may improve model prediction is moral norm. The construct represents beliefs about the behavior as being socially or culturally acceptable, perceiving a sense of social pressure to perform the behavior, and having a personal commitment to uphold the moral standards of their social group (Manstead, 2000). When controlling for the theory of planned behavior variables, moral norm has been shown to account for a small but significant improvement in the prediction of intention (Rivis et al., 2009). In the context of COVID-19, moral norm may be of particular relevance, given the strong value placed on complying with COVID-19 preventative behaviors by governments and health officials as the right thing to do or as part of citizens civic duty (Six et al., 2021). This is reflected in recent COVID-19 literature, as intention to follow social distancing and vaccination acceptance were predicted by the endorsement of those behaviors as the morally correct course of action (Anraad et al., 2020; Hagger et al., 2020).

Anticipated regret, an affective construct centered on the belief that engaging or not engaging in a behavior would result in significant negative consequences (Abraham and Sheeran, 2003), may also be useful to investigate in the COVID-19 context. Prior research has shown anticipated regret to be associated with a range of health behaviors (Modecki et al., 2022; Sandberg and Conner, 2008). In regard to COVID-19 preventative behaviors, anticipated regret has been theorized to be a potentially important behavioral determinant, speculating that individuals choosing not to follow recommended infection control behaviors could result in them becoming infected with COVID-19 or unwittingly spreading the virus to others and thus feeling regret, an unpleasant emotion that could have been avoided (Hagger et al., 2020; Smith et al., 2022).

A further point of the theory of planned behavior, and indeed social cognition models in general, is that intention does not always translate into actual behavior (Sheeran, 2002). A proposed explanation for this is that not all behaviors are enacted as the result of the deliberate decision-making process. For example, the steps of going to the store to get a loaf of bread and ensuring check-in with the QR code before entering the store are unlikely to require significant deliberation. Instead, it is likely many frequent and simple day-to-day behaviors, especially those that occur in regular contexts, are governed by highly efficient, automatic processes like *habit*, assessed most commonly as the extent to which an individual views their behavior as automatic and activated without conscious thought. In terms of general health behavior, habit has shown consistent effects on behavior, beyond those effects already accounted for by intention (Hagger et al., 2023; Phipps et al., 2020; Sas et al., 2023); an effect also demonstrated in COVID-19 preventive behaviors such as hand washing, facial mask wearing, avoiding crowds (Chang et al., 2022; Ohtomo and Kimura, 2022), and physical distancing (Hagger et al., 2022). This is in line with theory, as habit is expected to have particularly strong effects in consistent and stable contexts (Verplanken, 2006), and many COVID-19 preventive behaviors require consistent and regular action (e.g. putting on a mask whenever leaving the house; checking in at every venue; leaving 1.5 m gap every time you are in a queue), as is the case for the behavior in this study—QR code check-in compliance behavior.

## The present study

The aim of the present study was to identify the determinants of participation in QR code check-in compliance behavior among individuals in the context of COVID-19 using an integrated social cognition model that incorporated constructs from the Theory of Planned Behavior with moral norm, anticipated regret, and self-reported habit. Further, to allow for more nuanced findings, we tested this model in two Australian states which had differing experiences of the COVID-19 pandemic. That is, at the time of data collection, Victoria had experienced seven lockdowns, and the state capital, Melbourne, had the longest cumulative time in lockdown in the world (ABC News, 2021b). In contrast, after the initial pandemic wave, Queensland experienced a low rate of community transmission, and implemented only brief lockdown periods (three 3-day lockdowns, one 9-day lockdown), each of which was limited to local government areas in which community COVID-19 transmission had been detected or was considered likely. Thus, beyond the testing of an integrated social cognition model, we also aimed to explore for any differences in findings between the states to shed light on how factors influencing compliance behavior might differ based on the unique circumstances, regulations, and perceptions related to the pandemic in each state.

The hypotheses of this study are provided below, and also presented in Supplemental material as a Figure (see Appendix A). It was expected in both the Victoria and Queensland samples that:

H1: Attitude (H1a), subjective norm (H1b), perceived behavioral control (H1c), moral norm (H1d), anticipated regret (H1e), and baseline habit (H1f) would predict intention.
H2: Intention (H2a) and perceived behavioral control (H2b) would predict behavior.
H3: Attitude (H3a), subjective norm (H3b), perceived behavioral control (H3c), moral norm (H3d), anticipated regret (H3e), and baseline habit (H3f) would indirectly predict behavior via intention.
H4: Baseline habit would predict prospectively measured habit (H4a), and prospectively measured habit would, in turn, predict behavior (H4b).

## Method

### Participants and procedures

The study adopted a two-wave correlational design, with data collected between February 21st and March 28th, 2022. At this time, most people living in Australia had been vaccinated against COVID-19, and Governments were attempting to re-open large parts of the economy while minimizing additional COVID-19 cases. This required individuals to continue to log their entrance and provide proof of vaccination when entering hospitality and entertainment venues such as restaurants, cafés, bars, cinemas, nightclubs, and concert halls in both Victoria and Queensland. At the baseline time point, a sample of 580 Australian residents from Victoria (*N* = 290, 53.4% female) and Queensland (*N* = 290, 46.6% female) were recruited via an online research panel company using email and phone contacts. Participants were asked to provide informed consent, provided a definition of QR code check-in compliance behavior, and then asked to complete self-reported measures of theory of planned behavior constructs, moral norm, anticipated regret, and habit from the proposed integrated model (see Supplemental Material Appendix B for the full definition of QR code check-in compliance behavior and all self-report measures). Two weeks later, participants were recontacted via the panel company to complete follow-up measures of QR code check-in compliance behaviors and habit. From the initial sample, 128 participants from Queensland and 109 participants from Victoria did not return to complete follow-up measures, resulting in a final sample of 162 from Queensland (*M*_Age_ = 43.26, 47.5% female) and 181 from Victoria (*M*_Age_ = 41.88, 56.4% female). Participants received a small financial incentive for their participation based on expected completion time consistent with the panel company’s published rates. Approval for study procedures was granted prior to data collection from the Griffith University Human Research Ethics Committee. Full demographic information for the baseline and final samples in Victoria and Queensland is presented in supplemental material alongside attrition analyses (see Appendix C).

### Measures

Prior to completing the measures, participants were provided a definition of QR code check-in and compliance requirements, which involved the scanning of a QR code with a mobile device when entering hospitality or entertainment venues to aid contact tracing efforts in the context of COVID-19. Measures used were adopted from TPB recommendations (Ajzen, 2006) and adapted to the target behavior in the present study.

#### Attitude

Attitude toward participating in QR code check-in compliance behavior over the next 2 weeks was assessed using a three-item semantic differential scale. Each item was preceded with the common stem “Following COVID-19 QR code check-in and reporting compliance behaviors every time you enter a venue that requires you to check-in in the next 2 weeks would be. . .,” scored on a 7-point semantic differential scale (e.g. [1] Harmful to [7] Beneficial).

#### Subjective norm

Participants’ subjective norm to participate in QR code check-in compliance behavior over the next 2 weeks was assessed using a four-item scale (e.g. “Those people who are important to me would want me to follow COVID-19 QR code check-in and reporting compliance behaviors every time I enter a venue that requires me to check-in.”), with each item scored on a 7-point scale anchored [1] Strongly Disagree to [7] Strongly Agree.

#### Perceived behavioral control

Participants’ perceived behavioral control to participate in QR code check-in compliance behavior over the next 2 weeks was assessed using a four-item scale (e.g. “It is mostly up to me whether I follow COVID-19 QR code check-in and reporting compliance behaviors every time I enter a venue that requires me to check-in.”), with each item scored on a 7-point scale anchored [1] Strongly Disagree to [7] Strongly Agree.

#### Moral norm

Participants’ moral norm to follow in QR code check-in compliance behavior over the next 2 weeks was assessed using a three-item scale (e.g. “It is the right thing to do to follow COVID-19 QR code check-in and reporting compliance behaviors every time I enter a venue that requires me to check-in.”), with each item scored on a 7-point scale anchored [1] Strongly Disagree to [7] Strongly Agree.

#### Anticipated regret

Participants’ anticipated regret around not following QR code check-in compliance behavior over the next 2 weeks was assessed using a three-item scale (e.g. “If I did not follow COVID-19 QR code check-in and reporting compliance behaviors every time I enter a venue that requires me to check-in, I would feel regret.”), with each item scored on a 7-point scale anchored [1] Strongly Disagree to [7] Strongly Agree.

#### Intention

Intention to participate in QR code check-in compliance behavior over the next 2 weeks was assessed using a three-item scale (e.g. “I intend to follow COVID-19 QR code check-in and reporting compliance behaviors every time I enter a venue that requires me to check-in.”), with each item scored on a 7-point scale anchored [1] Strongly Disagree to [7] Strongly Agree.

#### Habit

Habit was assessed using the four item self-reported behavioral automaticity index (Verplanken and Orbell, 2003) (e.g. “Following COVID-19 QR code check-in and reporting compliance behaviors every time I enter a venue that requires me to check-in is something I do without having to consciously remember”), with each item scored on a 7-point scale anchored [1] Strongly Disagree to [7] Strongly Agree.

#### Demographic variables

Participants self-reported their age in years, gender, employment status (full time work, part-time/casual work, full time student, part-time student, unemployed, retired), marital status (married registered, married de facto, widowed, divorced, separated, never married), family taxable income (by range stratified by five income levels based on Australian national average), highest educational achievement (year 10, year 12, TAFE certificate/diploma, undergraduate degree, postgraduate degree).

### Data analysis

The data were analyzed as a linear multi-group partial least squares structural equation model (PLS-SEM) in WarpPLS 8.0, an alternative to traditional maximum likelihood SEM that is appropriate for exploratory research with modest samples and in contexts where non-normal data may be expected (Hair et al., 2017). Power analysis indicated a minimum required sample of 98 participants per state to achieve a power of 0.80, assuming modest effect sizes (*f*^2^ = 0.15). Model quality was assessed using the Tenenhaus goodness-of-fit index (GoF; acceptable if >0.36), the Simpsons paradox ratio (SPR; acceptable if >0.70), and the average variance inflation factor (AVIF; acceptable if <3.30). To ensure the robustness of our findings, standard errors were calculated using the stable method (Kock, 2018), a technique with results akin to bootstrapping while more robust to outliers and distributional problems. Parameter estimates were then compared between states using unequal variance assumed *t*-tests (Schenker and Gentleman, 2001), a statistical method that accounts for differences in sample size and variance between groups. To control family-wise error, *p*-values were adjusted using the Sidak adjustment, which is a conservative method for maintaining the overall type I error rate at a desired level.

## Results

**Figure 1.**
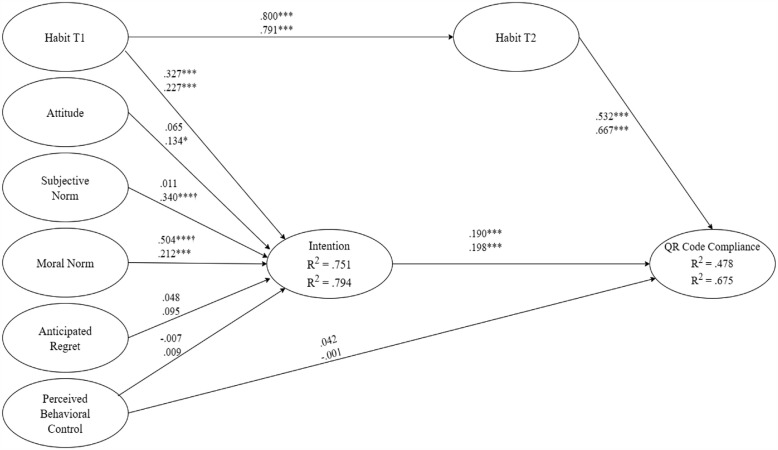
The model predicting QR code check-in compliance presented with standardized parameter estimates. Standardized parameter estimates of the integrated model. Coefficients printed on the upper line are for the Queensland sample and coefficients printed on the lower line are for the Victoria sample. † Effect of subjective norm on intention was significantly stronger in the Victoria sample. † Effect of moral norm on intention was significantly stronger in the Queensland sample. *p < 0.05. **p < 0.01. ***p < 0.001.

Descriptive statistics, zero-order correlations, and reliability statistics are presented in the online supplementary material (Appendix D). The model showed good fit-to-data in both Queensland (GoF = 0.737, SPR = 0.900, AVIF = 2.381) and Victoria (GoF = 0.786, SPR = 0.900, AVIF = 2.518) samples, and predicted a modest portion of variance in both intention (Queensland *R*^2^ = 0.751; Victoria *R*^2^ = 0.794) and COVID-19 check-in compliance behavior (Queensland *R*^2^ = 0.478; Victoria *R*^2^ = 0.675). Parameter estimates and difference statistics between samples are presented in Table 1. Intention predicted behavior in both samples (H2a-supported), while perceived behavioral control predicted behavior in neither (H2b-rejected). Intention to comply with COVID-19 check-in requirements was in turn predicted by baseline habit (H1f-supported) and moral norm (H1d-supported) in both samples. Moral norm also had a significant indirect effect on behavior via intentions in the Queensland sample (H3d-supported). Perceived behavioral control (H1c-rejected) and anticipated regret (H1e-rejected) did not predict intention in either sample. Subjective norm (H1b-supported) and attitude (H1a-supported) were predictive of intention in the Victoria sample, but not in the Queensland sample (H1a&b-rejected). Habit at the initial time point predicted habit at follow-up in both samples (H4a-supported), and habit at follow-up in turn predicted behavior (H4b-supported). Thus, baseline habit also had a significant indirect effect on behavior via follow-up habit. Comparison of parameter estimates between groups showed the effect of subjective norm on intention was significantly stronger in the Victoria sample, while the effect of moral norm was significantly stronger in the Queensland sample. No other parameter estimates significantly differed between samples. The model is presented visually in Figure 1.

**Table 1.** Standardized parameter estimates and difference statistics for the model predicting QR code check-in compliance in Queensland and Victoria.

	Queensland		Victoria		Diff. *d*	*p* Diff
	β	*p*	β	*p*		
Direct paths						
Habit T1 → Habit T2	0.800***	<0.001	0.791***	<0.001	0.019	1.000
Habit T1 → Intention	0.327***	<0.001	0.227***	<0.001	0.215	0.995
Attitude → Intention	0.065	0.153	0.134*	0.014	−0.148	1.000
Subjective Norm → Intention	0.011	0.432	0.340***	<0.001	−0.408	0.004**
Moral Norm → Intention	0.504***	<0.001	0.212***	<0.001	0.362	0.017*
Anticipated Regret → Intention	0.048	0.225	0.095	0.060	−0.058	1.000
Perceived Behavioral Control → Intention	−0.007	0.456	0.009	0.439	−0.020	1.000
Habit T2 → Behavior	0.532***	<0.001	0.667***	<0.001	−0.167	0.909
Intention → Behavior	0.190**	0.002	0.198***	<0.001	−0.010	1.000
Perceived Behavioral Control → Behavior	0.042	0.254	−0.001	0.491	0.053	1.000
Indirect paths						
Habit T1 → Habit T2 → Behavior	0.426***	<0.001	0.528***	<0.001	−0.178	0.856
Habit T1 → Intention → Behavior	0.062	0.083	0.045	0.149	0.030	1.000
Habit T1 (Total) → Behavior	0.488***	<0.001	0.573***	<0.001	−0.105	0.999
Attitude → Intention → Behavior	0.012	0.391	0.027	0.269	−0.026	1.000
Subjective Norm → Intention → Behavior	0.002	0.482	0.067	0.060	−0.114	0.998
Moral Norm → Intention → Behavior	0.096*	0.017	0.042	0.165	0.094	1.000
Anticipated Regret → Intention → Behavior	0.009	0.419	0.019	0.332	−0.017	1.000
Perceived Behavioral Control → Intention → Behavior	−0.001	0.488	0.002	0.483	−0.005	1.000

* *p* ⩽ 0.050. **p = 0.010. ***p < 0.001.

## Discussion

The Australian government has mandated QR code check-ins as part of its public health response to the COVID-19 pandemic, representing a novel approach to controlling infectious disease that has not been widely implemented elsewhere. Understanding the factors that determine compliance with these mandates is crucial for informing future public health interventions and preparedness efforts. To this end, this study employed an integrated social cognition model to identify the determinants of QR code check-in compliance behavior, supplementing the theory of planned behavior with moral norm and anticipated regret as additional predictors of intention, and habit as a determinant of behavior. The results indicated that intention (H2a) and habit (H4) significantly predicted QR code check-in compliance behavior in both Queensland and Victoria samples, with baseline habit (H1f) and moral norm (H1d) emerging as the most robust predictors of compliance intentions. Differences were also observed across the samples, as subjective norm was a stronger predictor of intention in Victoria than Queensland, while moral norm was a stronger predictor of intention in Queensland than Victoria. These findings have important implications for understanding compliance with public health mandates and for designing effective behavioral interventions to control infectious disease.

Somewhat in line with proposed predictions of the theory of planned behavior (Ajzen, 1991), intention (H2a), but not perceived behavioral control (H2b), was a significant predictor of QR code check-in compliance behavior in both samples. The observed lack of significant association between perceived behavioral control (H3c) and QR code check-in compliance behavior in both samples could be attributed to the perceived ease or simplicity of the behavior. The theory of planned behavior posits that perceived behavioral control is more salient in determining behavior when the behavior is perceived to be difficult or challenging (Ajzen, 1991). In instances where the behavior is not considered to be difficult, as is likely in the case of QR code check-in compliance behavior, perceived behavioral control may not exert a significant influence on behavior.

In regard to predicting intention with theory of planned behavior constructs, neither attitude (H1a), subjective norm (H1b), nor perceived behavioral control (H1c) predicted intention in the Queensland sample, and attitude (H1a) and subjective norm (H1b), but not perceived behavioral control (H1c), predicted intention in the Victoria sample. These results are in contrast with previous meta-analytic studies of the theory of planned behavior, where all model constructs have been supported as significant determinants of behavioral intentions for COVID-19 mitigation behaviors (Fischer and Karl, 2022) The finding that attitude (H1a) and subjective norm (H1b) predicted intention in the Victoria sample, but not in the Queensland sample, suggests that contextual factors may play a significant role in shaping the predictive utility of these theory of planned behavior constructs. In the context of the current study, it is worth considering the impact of experiential factors related to the COVID-19 pandemic in each state, such as the frequency and severity of lockdowns in Victoria compared to Queensland. For example, meta-analysis has indicated that populations with an increased emphasis and value on collective action have reported stronger effects of all theory of planned behavior constructs, with the most notable differences observed for the effects of subjective norms (Fischer and Karl, 2022). Given Victoria has experienced more extensive and prolonged lockdowns than Queensland, it is plausible that Victorians may have a greater awareness of and belief in the collective value of QR code check-ins as a public health measure, thereby influencing the salience of attitudes (H3a) and subjective norms (H3b) toward the behavior among the Victoria sample. In contrast, the lower incidence and severity of lockdowns in Queensland may have resulted in less urgency and lower perceived importance of public health measures such as QR code check-ins, thus reducing the predictive power of attitudes and subjective norms in shaping intentions and behavior. These findings highlight the importance of considering multiple contextual factors when examining the theory of planned behavior model and its applicability to different populations and settings.

Also, in contrast to hypotheses, anticipated regret (H1e) was not associated with intention to comply with COVID-19 check-in requirements in either sample. While this is unexpected given the likely regrettable consequences of contracting or spreading the COVID-19 virus, it is in line with previous COVID-19 research that has found weak or even null effects of anticipated regret on social distancing (Hagger et al., 2020) and hand hygiene (Smith et al., 2022). These results suggest that anticipated negative consequences may not play a major role in forming intentions to comply with COVID-19 preventive behaviors, and in the context of this study QR code check-in compliance behavior. Alternatively, the nature of COVID-19 preventive behaviors may lend themselves to other emotion-based influences rather than anticipated regret. For example, guilt (i.e. failing to live up to one’s own moral standards or the expectations of others) (Kugler and Jones, 1992), has been flagged as a potentially important predictor of prosocial behavior (Xu and Guo, 2018). Thus, it is possible guilt may be a more salient affective construct in the context of COVID-19, given the potential emotions elicited when individuals feel that they have put themselves or others at risk of contracting the virus by not adhering to preventive behaviors. In light of the unexpected null effects of anticipated regret observed in the current study and elsewhere (Hamilton et al., 2021), the investigation of alternative emotional drivers of infection control compliance likely warrants further research.

The present study found that moral norm (H1d) was a predictor of intention to comply with COVID-19 check-in requirements in both samples, although with a stronger effect in Queensland. The robustness of this effect across two different populations suggests that moral beliefs about the importance of complying with public health measures played a key role in shaping individuals’ intentions to follow QR code check-in requirements. This consistent effect may be explained considering the public health messaging presented by governments and health groups in both Queensland and Victoria throughout the pandemic. For example, numerous health messages and campaigns in both states focused on QR code check-ins as a core means to control the spread of COVID-19, safeguarding one’s own health and that of their community, and as a shared responsibility and opportunity for solidarity in the face of a common threat. Although additional research is required, these factors likely contributed to the consistency of the moral norm effect observed in the present study and may be indicative of the success of these public health campaigns.

Also, in addition to the social psychological constructs investigated that assess more deliberative decision-making processes, findings showed significant effects of habit on behavior, in that baseline habit predicted follow-up habit in both samples (H4a), and follow-up habit in turn predicted behavior over and above the effect of intention (H4b). This result is consistent with similar findings in a variety of other behaviors (Gardner, 2015; Hagger et al., 2023; Phipps et al., 2023), including COVID-19 preventative behaviors (Chang et al., 2022; Hagger et al., 2022; Ohtomo and Kimura, 2022). This adds to the body of literature supporting habit as a method of representing the automatic drivers on behavior that exist beyond deliberative, conscious decisions represented by intention. In the context of infection control strategies like QR code check-ins, these findings indicate frequently performed behaviors may eventually fall under the control of automatic cue-behavior scripts rather than effortful decision-making systems. Thus, the observed strong effects of habit may be indicative of the potential efficacy of habit development interventions on behaviors that are inherently repetitive and frequently undertaken. Importantly, this likely applies not only in the context of infection control behaviors as in the current study, but also in regard to numerous other health behaviors which are frequently undertaken, such as hand washing and sunscreen application. In such cases of frequent and stable behaviors, interventions aiming to promote the development of automatic cue-behavior scripts, such as providing nudges or promoting cue awareness, may be particularly efficacious for both encouraging and sustaining meaningful behavior change. However, additional empirical research is needed to verify the effectiveness of these strategies.

This research has several notable implications, both theoretical and practical. First, while the theory of planned behavior has been used extensively to predict COVID-19 preventive behaviors, this is the first study to use the model in the context of a new and novel COVID-19 preventive behavior; QR code check-in behavior. While large-scale contact tracing strategies are no longer a core element in mitigating the SARS-CoV-2 in many countries, this research presents a valuable perspective for understanding the social psychological factors guiding compliance in a particular behavior—QR code check-ins. This study holds various significant ramifications as although the behavior was not widely implemented globally, it was considered effective in curbing infection rates (ZDNET, 2020) and thus can help inform future public health messaging strategies for potential new COVID-19 variants or other health emergencies. In particular, normative expectations (i.e. approval from others to do the behavior) and moral obligations seem important to increase compliance for QR code check-in requirements. Thus, messaging could focus on fostering perceptions of social correctness and responsibility.

Of note, multi-group analysis indicated moral norm and subjective norm to be a stronger predictor in the Queensland and the Victoria sample, respectively. Findings could be interpreted considering the different experiences with the COVID-19 pandemic between the states. That is, while Queensland experienced only a small number of relatively short lockdowns and low infection rate throughout the pandemic, Victoria experienced multiple and extended lockdowns, including what was considered the world’s longest continuous lockdown (ABC News, 2021a), often paired with comparatively high rates of infection (ABC News, 2021a). It is plausible, therefore, that subjective norm (H1b) had strong effects on intention to comply with the QR code check-in system in Victoria given the virus posed a more immediate, proximal threat, and failure to comply would result in further long-term lockdowns, and thus, social pressures from important others to adhere to recommendations may have been particularly salient. In contrast, in Queensland, the low case rate and focus on a zero-COVID approach for much of the pandemic may have encouraged broader moral considerations of correctness and community responsibility (e.g. preventing lockdowns, keeping businesses open, avoiding overwhelmed hospitals) for complying with check-in requirements. These speculations need further investigation to be confirmed, including in depth qualitative data to fully understand the reasoning behind the differences between states.

Current findings also showed that complying with QR code check-in requirements has a component where the behavior is likely to be governed by automatic, nonconscious behavior. This is in line with findings of habit as a predictor of other COVID-19 preventive behaviors (Hagger et al., 2022; Harvey et al., 2021; Ohtomo and Kimura, 2022), and provides converging evidence for the value of using strategies to build habits targeting for infection control behaviors. Given the repetitiveness of mandated preventive behaviors like QR code check-in, engaging in this simple behavior may become habitual in a relatively short time span (Lally et al., 2010). In terms of pandemic management, the cumulative findings on the role of habit in COVID-19 prevention, in conjunction with habit theory, demonstrate the value of strategies to assist in habit development paired with the introduction of infection control strategies. Some common habit-forming strategies include setting specific goals or intentions (Hagger, 2019; Webb et al., 2009), using reminders or prompts (Andreassen et al., 2020), and practicing visualization or mental rehearsal (Webb et al., 2009).

### Strengths, limitations, and future directions

Major strengths of this study included the use of a prospective design, recruitment of a community sample across two Australian states who had different COVID-19 experiences, and adoption of an integrated theoretical model to identify behavioral determinants. Current results, however, should be considered in light of several limitations. We observed a relatively high attrition rate in both samples. While this is a potential threat to the validity of the research, attrition analysis showed minimal differences between those who completed the follow-up measures as compared to those who did not. Thus, it is unlikely the observed attrition caused serious bias in the findings but remains an important consideration in interpreting current results. The interpretation of data in the current study is grounded solely in theory, and the prospective design employed does not allow for empirical tests of causation or directionality. Given the use of QR code check-ins had been mandated for several months at the time of data collection, it is possible that the determinants of check-in compliance may have evolved over the pandemic due to factors like changing beliefs or “behavior fatigue” (Michie et al., 2020), and the factors which guided early adoption of QR code check-ins may differ from those observed in the current research. Further, QR code check-in compliance was assessed using self-reported measures. While previous research has supported the validity of similar brief self-reported behavioral measures (Hagger et al., 2020), it would be prudent for future research to consider replicating the current findings with more objective or observational measures of behavior.

## Conclusion

The aim of this research was to examine the determinants of a novel COVID-19 preventive behavior—QR code check-in compliance behavior—in samples from two Australian states, Queensland and Victoria, during the pandemic. Findings identified baseline habit, attitude, moral norm, and subjective norm predicted intention in the Victoria sample, and baseline habit and moral norm predicted intention in the Queensland sample. Intention and habit predicted behavior in both samples. This knowledge highlights targets for future intervention development and messaging aimed at increasing adherence to COVID-19 preventive behaviors, for example by framing compliance measures, such as QR code check-ins, as the “right thing to do” and encouraging habit formation toward compliance.

## Supplemental Material

sj-docx-1-hpq-10.1177_13591053231209880 – Supplemental material for Using an integrated social cognition model to identify the determinants of QR code check-in compliance behaviors in the COVID-19 pandemic Supplemental material, sj-docx-1-hpq-10.1177_13591053231209880 for Using an integrated social cognition model to identify the determinants of QR code check-in compliance behaviors in the COVID-19 pandemic by Thi Nhung Mac, Daniel J Phipps, Joy Parkinson, Mandy Cassimatis and Kyra Hamilton in Journal of Health Psychology

sj-pdf-2-hpq-10.1177_13591053231209880 – for Using an integrated social cognition model to identify the determinants of QR code check-in compliance behaviors in the COVID-19 pandemic sj-pdf-2-hpq-10.1177_13591053231209880 for Using an integrated social cognition model to identify the determinants of QR code check-in compliance behaviors in the COVID-19 pandemic by Thi Nhung Mac, Daniel J Phipps, Joy Parkinson, Mandy Cassimatis and Kyra Hamilton in Journal of Health Psychology This article is distributed under the terms of the Creative Commons Attribution 4.0 License ( http://www.creativecommons.org/licenses/by/4.0/) which permits any use, reproduction and distribution of the work without further permission provided the original work is attributed as specified on the SAGE and Open Access pages ( https://us.sagepub.com/en-us/nam/open-access-at-sage).
