# Supplementary material for: Using an integrated social cognition model to identify the determinants of QR code check-in compliance behaviors in the COVID-19 pandemic
Source: J Health Psychol. 2023 Nov 8;29(6):495–509. doi: 10.1177/13591053231209880 (PMC11075410; doi:10.1177/13591053231209880)
Supplement: sj-docx-1-hpq-10.1177_13591053231209880 – Supplemental material for Using an integrated social cognition model to identify the determinants of QR code check-in compliance behaviors in the COVID-19 pandemic [file sj-docx-1-hpq-10.1177_13591053231209880.docx]

**Appendix A**

Hypothesis - The Model Predicting QR Code Check-In Compliance presented with standardized parameter estimates


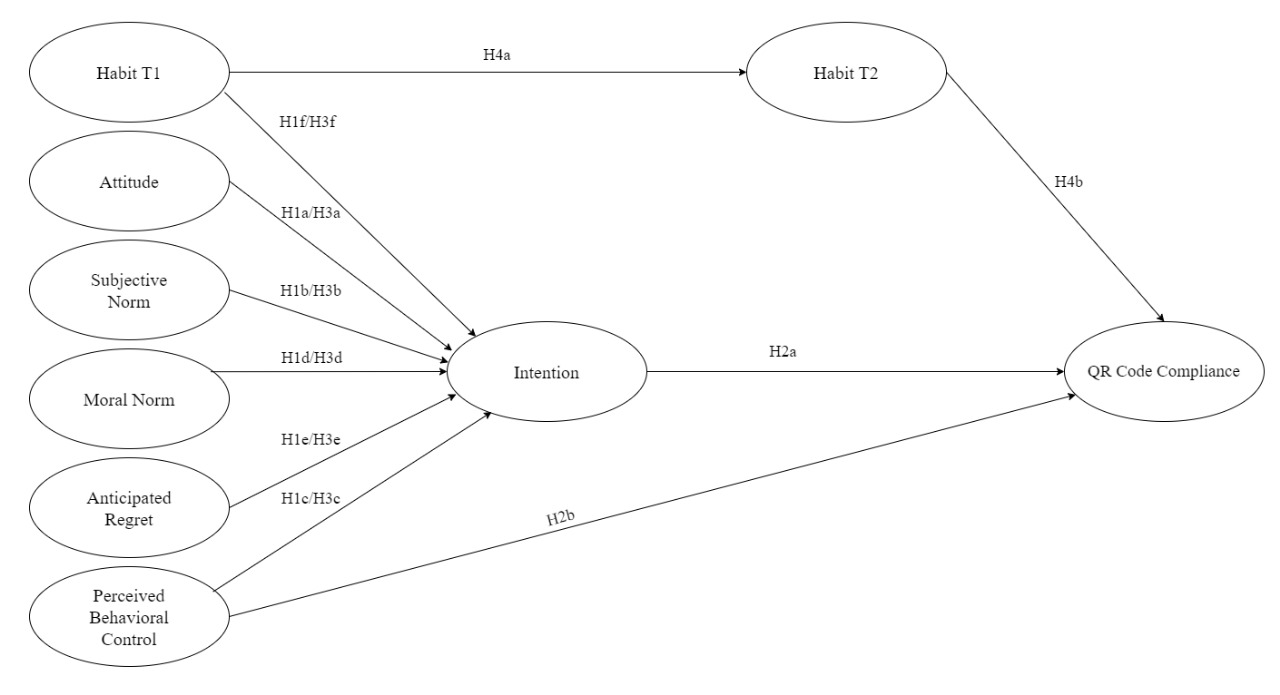


**Appendix B**

A QR code is a link that your smartphone camera can recognise. When you check-in, you create a record of the time and date you visited a venue or show evidence of your vaccination status. This means that if there is a COVID-19 outbreak, contact tracers can quickly access your contact information and get in touch with you if you have visited a public exposure site or venue staff can check that you are fully vaccinated.

*Items and Response Scales for Variables of the Integrated Model*

| Variable | Item(s)/measure | Scale |
| --- | --- | --- |
| Attitude | Following COVID-19 QR code check-in and reporting compliance behaviours every time you enter a venue that requires you to check-in in the next 2 weeks would be... | 1 = harmful, 7 = beneficial  1 = bad, 7 = good  1 = worthless, 7 = valuable |
| Subjective norm | How much do you agree with each of the below statements regarding following COVID-19 QR code check-in and reporting compliance behaviours every time you enter a venue that requires you to check-in in the next two weeks?  Those people who are important to me would want me to follow COVID-19 QR code check-in and reporting compliance behaviours every time I enter a venue that requires me to check-in. (1)  Other people I know follow COVID-19 QR code check-in and reporting compliance behaviours every time they enter a venue that requires them to check-in (2)  Most people who are important to me would approve of me following COVID-19 QR code check-in and reporting compliance behaviours every time I enter a venue that requires me to check-in. (3)  Most people who are important to me think I should follow COVID-19 QR code check-in and reporting compliance behaviours every time I enter a venue that requires me to check-in. (4) | 1 = strongly disagree, 7 = strongly agree |
| Moral norm | How much do you agree with each of the below statements regarding following COVID-19 QR code check-in and reporting compliance behaviours every time you enter a venue that requires you to check-in in the next two weeks?  It is the right thing to do to follow COVID-19 QR code check-in and reporting compliance behaviours every time I enter a venue that requires me to check-in. (1)  It is my moral responsibility to follow COVID-19 QR code check-in and reporting compliance behaviours every time I enter a venue that requires me to check-in. (2)  It is my moral obligation to follow COVID-19 QR code check-in and reporting compliance behaviours every time I enter a venue that requires me to check-in. (3) | 1 = strongly disagree, 7 = strongly agree |
| Anticipated regret | How much do you agree with each of the below statements regarding following COVID-19 QR code check-in and reporting compliance behaviours every time you enter a venue that requires you to check-in in the next two weeks?  If I did not follow COVID-19 QR code check-in and reporting compliance behaviours every time I enter a venue that requires me to check-in, it would upset me (1)  If I did not follow COVID-19 QR code check-in and reporting compliance behaviours every time I enter a venue that requires me to check-in, I would feel regret (2)  If I did not follow COVID-19 QR code check-in and reporting compliance behaviours every time I enter a venue that requires me to check-in, I would feel sorry for not doing it (3) | 1 = strongly disagree, 7 = strongly agree |
| Perceived behavioural control | How much do you agree with each of the below statements regarding following COVID-19 QR code check-in and reporting compliance behaviours every time you enter a venue that requires you to check-in in the next two weeks?  It is mostly up to me whether I follow COVID-19 QR code check-in and reporting compliance behaviours every time I enter a venue that requires me to check-in. (1)  I have complete control over whether I follow COVID-19 QR code check-in and reporting compliance behaviours every time I enter a venue that requires me to check-in. (2)  It would be easy for me to follow COVID-19 QR code check-in and reporting compliance behaviours every time I enter a venue that requires me to check-in. (3)  I am confident that I could follow COVID-19 QR code check-in and reporting compliance behaviours every time I enter a venue that requires me to check-in. (4) | 1 = strongly disagree, 7 = strongly agree |
| Intention | In the next 2 weeks…  It is likely that I will follow COVID-19 QR code check-in and reporting compliance behaviours every time I enter a venue that requires me to check-in. (1)  I intend to follow COVID-19 QR code check-in and reporting compliance behaviours every time I enter a venue that requires me to check-in. (2)  I plan to follow COVID-19 QR code check-in and reporting compliance behaviours every time I enter a venue that requires me to check-in. (3) | 1 = strongly disagree, 7 = strongly agree |
| Habit | Following COVID-19 QR code check-in and reporting compliance behaviours every time I enter a venue that requires me to check-in is something…  I do automatically (1)  I do without having to consciously remember (2)  I do without thinking (3)  I start to do it before I realize I’m doing it (4) | 1 = strongly disagree, 7 = strongly agree |
| Past behaviour/  behaviour | In the past two weeks, how often did you follow COVID-19 QR code check-in and reporting compliance behaviours every time you enter a venue that requires you to check-in?  In the past two weeks, to what extent did you follow COVID-19 QR code check-in and reporting compliance behaviours every time you entered a venue that requires you to check-in? | 1 = never, 7 = always.  1 = a small extent, 7 = a large extent |

**Appendix C**

From the initial sample, 128 participants from Queensland and 109 participants from Victoria did not return to complete follow-up measures, resulting in a final sample of 162 from Queensland (MAge= 43.26, 47.5% female) and 181 from Victoria (MAge= 41.88, 56.4% female). While there was higher than expected attrition at the follow-up data collection point, there was no significant difference in the response rate as a function of gender (Queensland χ^2^(2)=.97, *p* = .614; Victoria χ^2^(2)=.97, *p* = .305), employment status (Queensland χ^2^(4)= 2.80, *p* = .580; Victoria χ^2^(4)= 6.71, *p* = .243), education level (Queensland χ^2^(4)= 7.57, *p* = .109; Victoria χ^2^(4)= 5.79, *p* = .216), household income (Queensland χ^2^(4)= 7.68, *p* = .175; Victoria χ^2^(4)= 4.18, *p* = .523), ethnicity (Queensland χ^2^(2)= 1.85, *p* = .397; Victoria χ^2^(2)= 0.31, *p* = .855), or baseline study variable scores (Queensland λ=.04, *F*(6, 283)= 0.29, *p* = .941; Victoria λ=.96, *F*(6, 283)= 1.75, *p* = .109). However, there were significant effects of relationship status (Queensland χ^2^(5)= 12.81, *p* = .025; Victoria χ^2^(5)= 16.27, *p* = .006) and age (Queensland *t*(288)= 7.25, *p*< .001, *d*= 0.86; Victoria *t*(288)= 6.86, *p*< .001, *d*= 0.83), as participants who were older and had been married were more likely to return to complete follow-up measures.

*Sample Characteristics and Descriptive Statistics for Study Variables at Baseline and at 2-Week Follow-up*

| Variable | Baseline | | Follow-up | |
| --- | --- | --- | --- | --- |
|  | Queensland | Victoria | Queensland | Victoria |
| **Participants** | 290 | 290 | 162 | 181 |
| Age, M years (SD) | 38.99 (12.26) | 38.27 (12.42) | 43.26 (11.31) | 41.88 (11.51) |
| **Gender** |  |  |  |  |
| Male | 154 | 134 | 84 | 78 |
| Female | 135 | 155 | 77 | 102 |
| Non-binary / third gender | 1 | 0 | 1 | 0 |
| Prefer not to say | 0 | 1 | 0 | 1 |
| **Current relationship status** |  |  |  |  |
| Married registered | 95 | 82 | 59 | 59 |
| Married de facto | 60 | 48 | 31 | 33 |
| Widowed | 1 | 1 | 1 | 1 |
| Divorced | 16 | 17 | 14 | 11 |
| Separated | 6 | 8 | 4 | 8 |
| Never Married | 112 | 134 | 53 | 69 |
| **Employment status** |  |  |  |  |
| Full-time work | 166 | 144 | 90 | 87 |
| Part-time/Casual work | 62 | 76 | 32 | 47 |
| Full-time student | 9 | 11 | 5 | 5 |
| Part-time student | 0 | 3 | 0 | 1 |
| Unemployed | 35 | 42 | 23 | 29 |
| Retired | 18 | 14 | 12 | 12 |
| **Highest educational achievement** |  |  |  |  |
| Year 10 | 28 | 17 | 14 | 14 |
| Year 12 | 47 | 59 | 19 | 32 |
| TAFE certificate/diploma | 98 | 72 | 61 | 43 |
| Undergraduate degree | 77 | 96 | 47 | 60 |
| Postgraduate degree | 40 | 46 | 21 | 32 |
| **Family taxable income range** |  |  |  |  |
| Nil – $18,200 | 4 | 10 | 4 | 4 |
| $18,201 - $37,000 | 32 | 35 | 19 | 20 |
| $37,001 – $80,000 | 65 | 85 | 38 | 51 |
| $80,001 – $180,000 | 110 | 99 | 53 | 67 |
| >$180,001 | 54 | 31 | 31 | 19 |
| Prefer Not to Say | 25 | 30 | 17 | 20 |

| **Appendix D**  *Bivariate Correlations, Descriptive, and Reliability for Statistics for Demographic Variables and Constructs Used to Predict QR Code Check-in Compliance Behavior* | | | | | | | | | | | | | | | |
| --- | --- | --- | --- | --- | --- | --- | --- | --- | --- | --- | --- | --- | --- | --- | --- |
| Variables | | 1 | 2 | 3 | 4 | 5 | 6 | 7 | 8 | 9 | 10 | 11 | Mean | SD | Reliability |
| 1 | Age | - | -.312^**^ | .047 | -.006 | .073 | .002 | .004 | .063 | .032 | .101 | .045 | 38.99 | 12.26 | - |
| 2 | Gender | .004 | - | -.002 | .035 | -.006 | .097 | .075 | .048 | .015 | .148 | .061 | - | - | - |
| 3 | Habit T1 | .027 | .059 | - | .644^**^ | .665^**^ | .686^**^ | .633^**^ | .325^**^ | .713^**^ | .799^**^ | .497^**^ | 4.39 | 1.89 | .95 |
| 4 | Attitude T1 | .031 | .040 | .644^**^ | - | .738^**^ | .752^**^ | .677^**^ | .358^**^ | .683^**^ | .560^**^ | .484^**^ | 4.63 | 1.81 | .93 |
| 5 | Subjective norms T1 | -.013 | .040 | .668^**^ | .707^**^ | __ | .807^**^ | .629^**^ | .428^**^ | .758^**^ | .531^**^ | .390^**^ | 4.91 | 1.63 | .95 |
| 6 | Moral norms T1 | .015 | .109 | .606^**^ | .711^**^ | .796^**^ | - | .696^**^ | .438^**^ | .820^**^ | .634^**^ | .515^**^ | 4.87 | 1.82 | .96 |
| 7 | Anticipated regret T1 | -.017 | .042 | .608^**^ | .611^**^ | .653^**^ | .684^**^ | - | .334^**^ | .631^**^ | .582^**^ | .477^**^ | 3.46 | 1.87 | .95 |
| 8 | PBC T1 | .021 | -.025 | .465^**^ | .528^**^ | .531^**^ | .556^**^ | .362^**^ | - | .368^**^ | .299^**^ | .262^**^ | 5.37 | 1.18 | .71 |
| 9 | Intention T1 | -.019 | .028 | .734^**^ | .731^**^ | .801^**^ | .802^**^ | .696^**^ | .551^**^ | - | .680^**^ | .565^**^ | 4.86 | 2.01 | .98 |
| 10 | Habit T2 | .023 | -.002 | .792^**^ | .639^**^ | .650^**^ | .618^**^ | .650^**^ | .513^**^ | .731^**^ | - | .675^**^ | 4.11 | 1.98 | .97 |
| 11 | Behavior T2 | .049 | .049 | .617^**^ | .575^**^ | .583^**^ | .548^**^ | .515^**^ | .458^**^ | .684^**^ | .811^**^ | - | 4.39 | 2.38 | .92 |
|  | Mean | 38.27 | - | 4.55 | 4.92 | 5.00 | 5.07 | 3.71 | 5.53 | 4.88 | 4.17 | 4.45 |  |  |  |
|  | Standard Deviation | 12.42 | - | 1.84 | 1.88 | 1.65 | 1.81 | 1.95 | 1.18 | 1.99 | 2.11 | 2.38 |  |  |  |
|  | Reliability | - | - | .95 | .94 | .95 | .97 | .97 | .77 | .98 | .97 | .93 |  |  |  |
| Note. Statistics above the diagonal are from the Queensland sample (*N* = 162); Statistics from below the diagonal refer to the Victoria sample (*N* = 290 in *N* = 181). PBC refers to perceived behavioral control. ** Correlation is significant at the 0.01 level (2-tailed) | | | | | | | | | | | | | | | |
