# Supplementary material for: Using an integrated social cognition model to identify the determinants of QR code check-in compliance behaviors in the COVID-19 pandemic
Source: J Health Psychol. 2023 Nov 8;29(6):495–509. doi: 10.1177/13591053231209880 (PMC11075410; doi:10.1177/13591053231209880)
Supplement: sj-pdf-2-hpq-10.1177_13591053231209880 – for Using an integrated social cognition model to identify the determinants of QR code check-in compliance behaviors in the COVID-19 pandemic [file sj-pdf-2-hpq-10.1177_13591053231209880.pdf]

**Supplementary Materials**

Supplementary Materials are available at https://osf.io/dxfm2/

Supplementary files include the following:

Data - Deidentified.sav: The dataset used in the current analysis, minus any identifying information

Log Files and Additional Analysis - State 1/2.txt: A log file of the output from WarpPLS 8.0. This contains the settings used in model analysis for replication, as well as all model output and additional analyses.

Supplementary Appendix A: Survey Materials.docx: A copy of all survey questions and their relevant scoring scales.

Note as WarpPLS 8.0 uses a graphical interface and does not allow for or produce log syntax, this information is not included.

Replication can be conducted by downloading the included dataset and running the analysis as detailed in the Log Files and Additional Analysis.txt files.
